# Cefdinir-Associated Myopericarditis

**DOI:** 10.7759/cureus.102427

**Published:** 2026-01-27

**Authors:** Chirag Lodha, Bharath Subramanian, Matthew Bajaj, Patrick J Rogers, Eric J Basile, Luke Casals, Krunal Shukla, Jonathan Van Name, Afrin Naz

**Affiliations:** 1 Internal Medicine, University of South Florida Morsani College of Medicine, Tampa, USA; 2 Medicine, USF Health, Tampa, USA; 3 Cardiovascular Disease, University of South Florida Morsani College of Medicine, Tampa, USA; 4 Internal Medicine, University of Florida College of Medicine, Gainesville, USA

**Keywords:** cardiology, cefdinir, drug-related side effects and adverse reactions, myopericarditis, pericarditis

## Abstract

Cefdinir is a third-generation cephalosporin with a wide variety of indications and a strong safety profile. A 24-year-old man began taking cefdinir for an uncomplicated urinary tract infection. Two days later, he began experiencing pleuritic, sharp chest pain and diarrhea and was shown to have mildly elevated troponins. Due to the pleuritic nature of his chest pain improving when leaning forward, as well as elevated troponins and a new small pericardial effusion on echocardiogram, he was diagnosed with myopericarditis thought to be secondary to cefdinir. Cefdinir was subsequently held with diarrhea and chest pain improving shortly afterwards. He was started on ibuprofen 600mg TID for two weeks, as well as colchicine 0.5mg daily for six weeks. The patient finished his treatment regimen and does not endorse recurrence of his chest pain or any symptoms of heart failure, such as orthopnea, lower extremity edema, or shortness of breath.

## Introduction

Cefdinir is a third-generation cephalosporin with a broad spectrum of bacterial coverage, including common pathogens such as *Streptococcus pneumoniae,* *Haemophilus influenzae*, and *Moraxella *[1]. It boasts a strong safety profile, with main side effects being GI-related (especially diarrhea) and very few documented cardiac effects [1]. 

Myocarditis is clinically defined as inflammation of the myocardium or heart muscle tissue. The disease can vary in clinical phenotype from mild chest pain to acute heart failure and shock. Most cases worldwide are caused by viruses, but drug toxicity can lead to a condition called hypersensitivity myocarditis with eosinophilic infiltration of the heart tissue as a result of an allergic reaction [1]. Hypersensitivity myocarditis is a rare complication of antibiotic use. It has been known to occur in the use of antipsychotics, immunotherapies, and vaccines but more rarely occurs in the use of antibiotics such as penicillins [1,2]. A few cases have been described in the literature related to cephalosporins, which are derivatives of penicillins. These cases have described the use of other cephalosporins such as cefaclor, cefepime, and cephalexin [3,4].

## Case presentation

**Figure 1:**
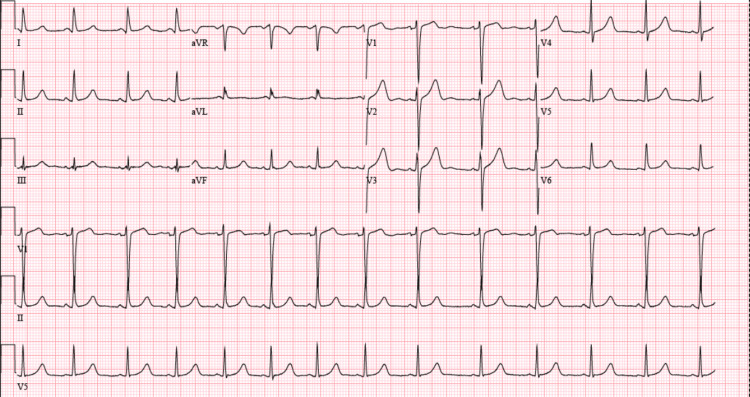
EKG showing diffuse ST-segment elevations

A 24-year-old man with a past medical history of depression and asthma presented to the emergency department with chest pain and pressure, along with elbow pain and diarrhea. He describes the pain as a heavy pressure in the center of his chest without radiation and notes it started after taking cefdinir for a urinary tract infection (UTI). He stated that the sensation would improve upon leaning forward and was worse when he was lying down. On review of systems, he had a rhinovirus upper respiratory infection (URI) one month prior. Regarding his diarrhea, he states 8 to 10 episodes daily of watery diarrhea and denied any blood or mucus in his stools. He states that this bilateral elbow joint pain and chest pain all seem to begin when starting his cefdinir course. His physical exam was unremarkable. No murmurs, rubs, or gallops were auscultated, and chest pain was non-reproducible with palpation. In the emergency department, his EKG showed a normal sinus rhythm with slight PR-interval depression with slight ST-elevations in leads V2-V4 (Figure 1). 

**Figure 2:**
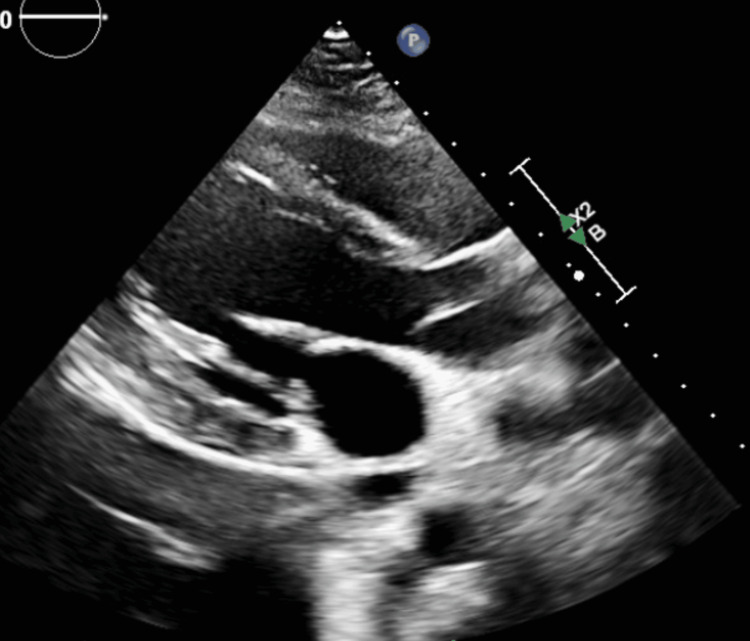
Echocardiogram showing trivial pericardial effusion

His chest X-ray was unremarkable for any acute processes. However, his troponin was initially high and subsequently increased three hours later. His B-natriuretic peptide was normal, C-reactive protein (CRP) was high, and erythrocyte sedimentation rate was high (Table 1). Echocardiogram showed no wall motion abnormalities, an ejection fraction of approximately 58%, and a small pericardial effusion (Figure 2).

**Table 1:** Laboratory values showing serial cardiac biomarkers and inflammatory markers during the patient's hospital course

Laboratory Test	Patient Value	Reference Range
Troponin (initial)	129 ng/L	< 35 ng/L
Troponin (three hours later)	312 ng/L	< 35 ng/L
Troponin (one day later)	199 ng/L	< 35 ng/L
B-type natriuretic peptide (BNP)	56 pg/mL	< 100 pg/mL
C-Reactive protein (CRP)	1.9 mg/dL	< 1 mg/dL
Erythrocyte sedimentation rate (ESR)	18 mm/hr	<15 mm/hr

Due to the positional nature of the patient's chest pain, mild PR-interval depression, and new small pericardial effusion, as well as elevated troponin, he was diagnosed with acute myopericarditis. Due to the coincident onset of symptoms with his UTI treatment, it was thought to be secondary to cefdinir. To confirm this, his cefdinir was discontinued, and his diarrhea, arthralgias, and chest pain quickly resolved, with troponin decreasing to 199 ng/L. He was started on ibuprofen 600mg TID for two weeks, as well as colchicine 0.5mg daily for six weeks. The patient finished his treatment regimen and does not endorse recurrence of his chest pain or any symptoms of heart failure such as orthopnea, lower extremity edema, or shortness of breath. 

## Discussion

Myocarditis is clinically defined as inflammation of the myocardium or heart muscle tissue. The disease can vary in clinical picture from mild chest pain to acute heart failure and shock. The leading cause of myocarditis is viruses, but drug toxicity can lead to a condition called hypersensitivity myocarditis with eosinophilic infiltration of the heart tissue as a result of a hypersensitivity reaction [1]. 

Hypersensitivity myocarditis is a rare complication of antibiotic use. It has been known to occur in the use of antipsychotics, immunotherapies, and vaccines but more rarely occurs in the use of antibiotics such as penicillins [1,2]. A few cases have been described in the literature relating to cephalosporins, which are derivatives of penicillins. These cases have described the use of other cephalosporins such as cefaclor, cefepime, and cephalexin [3,4]. The clinical presentation of hypersensitivity myocarditis is characterized by chest pain, acute rash, fever, and myalgia [5,6]. In this case, our patient presented with chest pain that began shortly after taking his cefdinir, along with bilateral elbow joint pain. He was afebrile throughout the course of his hospital stay and did not have a rash present on his body. In a previous study, only 35.5% of patients with histologically provable hypersensitivity myocarditis had a fever and only 22.3% of these patients also had nonspecific symptoms that we saw such as joint pain [6]. Previous history of asthma was also seen in 21.1% of patients in their study, which was in our patient’s medical history as well [6]. Notable lab values for hypersensitivity myocarditis on admission include peripheral eosinophilia (seen in 75.9% of patients) as well as increased troponins (95.7% of patients) and CRP (79.5% of patients) [6]. On admission, the patient had elevated troponin levels, with an initial value of 130 ng/L. His CRP was also elevated on admission at 1.9 g/L. Our patient, however, did not have eosinophilia, with an eosinophil count on admission of 16/mm^3^. EKG changes are common in hypersensitivity myocarditis, with only 9.4% of patients having a normal EKG [6]. Our patient was found to have an EKG with a normal sinus rhythm showing a very uncommon presentation. Around 34.1% of patients have a concurrent pericardial effusion; our echocardiograph showed a trivial concurrent pericardial effusion, increasing our suspicion of myopericarditis for this patient’s clinical presentation [6]. 

An additional consideration was the fact that the patient had a previous rhinovirus URI approximately one month before presentation, leading to the possibility of a rhinovirus-induced myocarditis. Although this is more common than an adverse effect of cefdinir, the patient’s Naranjo score, indicating the probability of an adverse drug reaction, was 6 [7]. This indicated a fairly high probability that his myocarditis was secondary to his UTI treatment with cefdinir. Typical treatment of hypersensitivity myocarditis involves the cessation of the inciting medication, in this case, cefdinir. Our patient's presentation was more similar to myopericarditis as he had chest pain that was worse with cough and improved with leaning forward with elevated troponins signifying myocardial inflammation. Guidelines state NSAIDs as the first-line treatment. Our patient was started on ibuprofen 600mg TID for two weeks. In addition to this, colchicine has shown some efficacy in myocarditis and with the presentation suggesting possible myopericarditis, he was started on 0.6mg of colchicine for six weeks [8]. He was also recommended to undergo cardiac MRI for a definitive diagnosis of myocarditis but had not done so by the time of writing. Severe cases of myocarditis are treated with therapy of the resulting sequelae. Arrhythmias are treated with pacemakers and possible ICDs on a case-by-case basis. Heart failure caused by myocarditis is treated according to current heart failure guidelines. Severe cases of hypersensitivity myocarditis can be treated with immunosuppression or steroids [8]. In our case, such drugs were avoided as our patient had a mild presentation of myocarditis, and the offending drug was ceased immediately before his condition could progress to heart failure. 

## Conclusions

In conclusion, hypersensitivity myocarditis should be suspected in the presence of new-onset chest pain and rising troponins after a recent medication change. This condition can possibly be caused by cephalosporin antibiotics. Other lab values, such as eosinophilia, may not be present in this condition. Other subjective criteria that are used to diagnose myocarditis such as fever and EKG changes may also not be noticeable. Prompt evaluation and cessation of inciting medication are necessary to prevent life-threatening arrhythmias and heart failure.

This case shows the importance of atypical causes of chest pain in young patients who present with laboratory evidence of myocardial injury. While cefdinir is a widely prescribed antibiotic with a strong safety profile, it can rarely cause cardiac complications. Recognizing medications that patients are on when they present to the hospital can speed up decision-making and treatment to reverse life-threatening conditions that are caused by these medications.
